# The Work and Needs of the Public Health Service

**Published:** 1915-02

**Authors:** 


### The Work and Needs of the Public Health Service.

The Public Health Service is coming into its own. Hampered as it was for many years as an appendage to the Marine Hospital Service, neither civil nor military, its work restricted by meager appropriations, it has, nevertheless, gone steadily forward under the able leadership of such men as Hamilton, Wyman and Blue, until by act of Congress it has now attained the dignity which is its due. It still holds the anomalous position of an appendage of the Treasury Department, but shares this misfortune with other valuable and extraneous services, like the Revenue Cutter Service, the Lighthouse Service and the Life Saving Service.

Notwithstanding its grievous limitations the United States Public Health Service, by the excellence of its work, has climbed steadily until it has now reached the top of the ladder of sanitary efficiency, and is known and respected the world over.

The report of the surgeon general for the year 1914 details very briefly and without flourish of trumpets the protean energies of this Service which is devoted not to the healing of the nation but to the protection from disease of those within its gates.

The list of the various activities of this bureau alone would be too long to enumerate here, but they may be grouped under the following seven divisions: (1) Scientific research and sanitation, including studies of beriberi, diphtheria, goiter, hookworm, smallpox, pellagra, trachoma, tuberculosis, occupational diseases and industrial hygiene, rural sanitation, etc.; (2) foreign and insular quarantine; (3) domestic or interstate quarantine; (4) sanitary reports and statistics; (5) marine hospitals and relief; (6) personnel and accounts, and (7) miscellaneous.

The overwhelming importance of the first division is shown by the amount of space, one hundred and eighty pages, devoted to it in the annual report which with the index comprises only three hundred and fifty-seven pages.

The personnel of the Service, while of most excellent quality, is far too small for the enormous work which comes under its supervision and care. During the last fiscal year twenty-three additional assistant surgeons were appointed, but notwithstanding this increase, the employment of 239 acting assistant surgeons was necessary.

An important factor in the educational propaganda conducted by the bureau is the weekly publication known as *Public Health Report,* which now has a circulation of about 11,000 copies. The five departments which make up this publication give the current prevalence and geographic distribution of the preventable and controllable diseases in the United States; sanitary legislation showing State laws and regulations; city ordinances and regulations and court decisions on matters pertaining to the public health; the prevalence and geographic distribution in foreign countries of diseases quarantinable in the United States; matters of interest to health officers, largely educational in nature and supplements which are issued as monographs relating to personal and community hygiene and intended for general distribution. Of these latter, ten appeared during the year on subjects varying from the summer care of infants to the treatment of syphilis and sanitary conditions among the Eskimos, interspersed with such lighter topics as shower baths for country houses and what the farmer can do to prevent malaria.

The needs of the service as outlined by the surgeon general are an increase in the number of trained officers for Federal public health work, additional clerical assistance in the bureau at Washington, an additional building for the use of the hygienic laboratory, an appropriation for the employment of persons in the collection of morbidity statistics, and the stimulation and encouragement of the educational propaganda for public health.

We said at the beginning, and advisedly, that the Public Health Service is coming into its own. It cannot arrive, however, in our opinion, until its independence is assured by its divorce from the Treasury Department and its establishment as an independent department of the government.—*The Military Surgeon.*

